# Supplementary material for: A Novel Anti-CD44 Variant 3 Monoclonal Antibody C44Mab-6 Was Established for Multiple Applications
Source: Int J Mol Sci. 2023 May 7;24(9):8411. doi: 10.3390/ijms24098411 (PMC10179237; doi:10.3390/ijms24098411)
Supplement: Supplementary file 1 [file ijms-24-08411-s001.zip › Supple Figs & Tables/Supple Fig. S1 S2 C44Mab-6 .pdf]

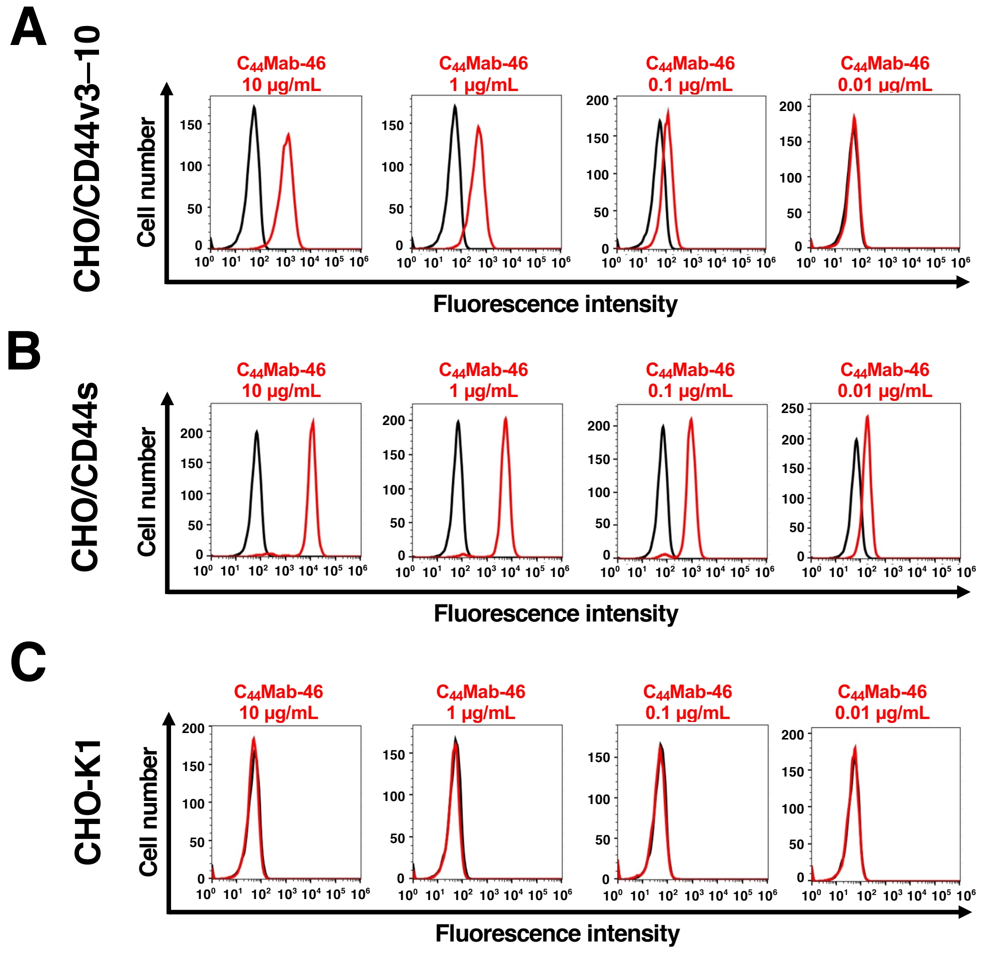

Figure S1 Recognition of CHO/CD44s and CHO/CD44v3–10 by C_{44}Mab-46 using flow cytometry. CHO/CD44v3–10 (A), CHO/CD44s (B), and CHO-K1 (C) were treated with 0.01-10 µg/mL of C_{44}Mab-46, followed by treatment with Alexa Fluor 488-conjugated anti-mouse IgG (Red line). The black line represents the negative control (blocking buffer).

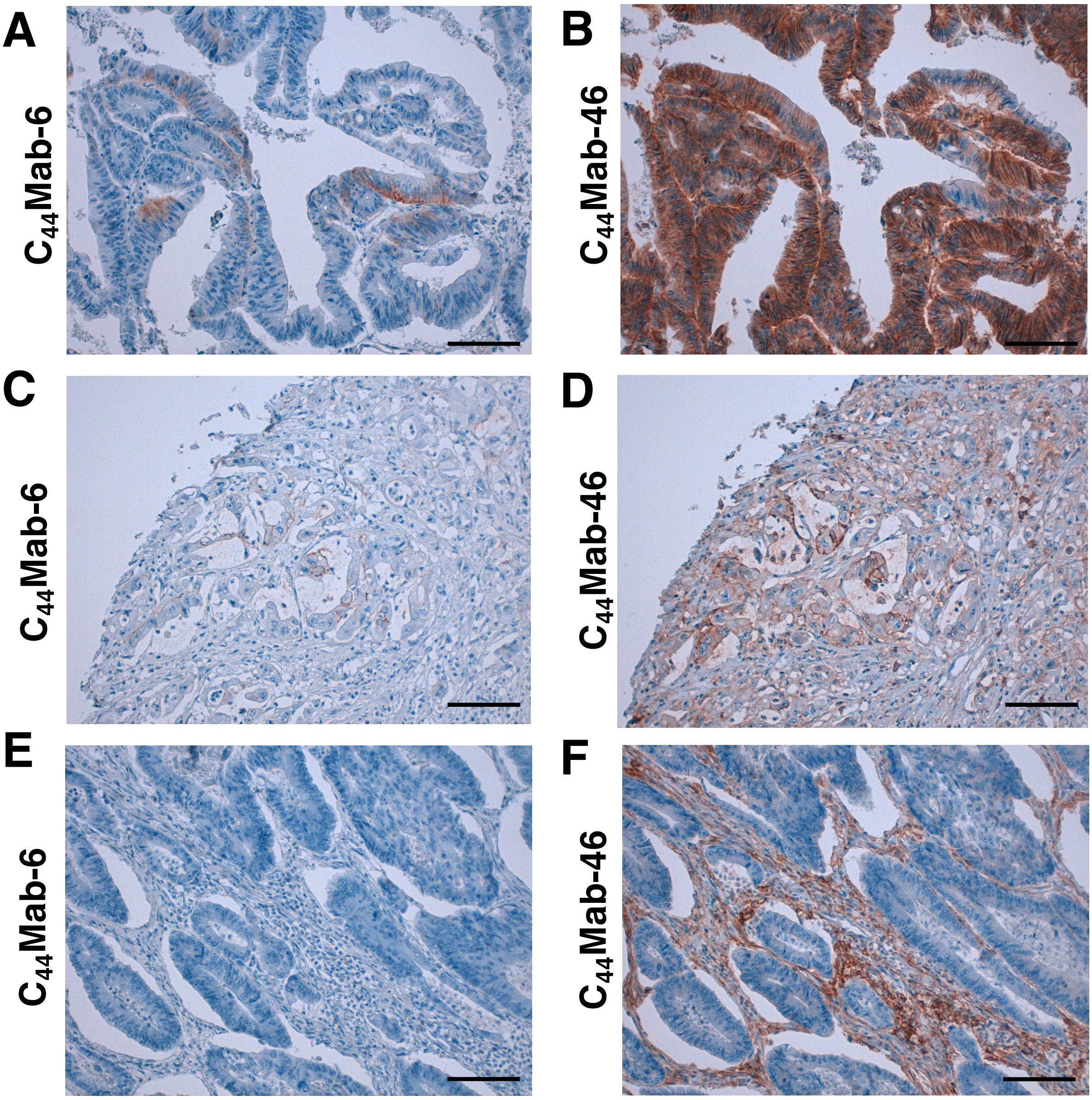

Figure S2. Immunohistochemical analysis using C_{44}Mab-6 and C_{44}Mab-46 against colorectal carcinoma tissues. After antigen retrieval, serial sections of colorectal carcinoma tissue arrays (CO483a) were incubated with 1 μg/mL of C_{44}Mab-6 (**A**,**C**,**E**) or C_{44}Mab-46 (**B**,**D**,**F**) followed by treatment with the Envision+ kit. The color was developed using 3,3′-diaminobenzidine tetrahydrochloride (DAB), and the sections were counterstained with hematoxylin. Scale bar = 100 μm.
