# Supplementary material for: A Novel Anti-CD44 Variant 3 Monoclonal Antibody C44Mab-6 Was Established for Multiple Applications
Source: Int J Mol Sci. 2023 May 7;24(9):8411. doi: 10.3390/ijms24098411 (PMC10179237; doi:10.3390/ijms24098411)
Supplement: Supplementary file 1 [file ijms-24-08411-s001.zip › Supple Figs & Tables/Supple Table S1, 2 C44Mab-6 .pdf]

Supplementary Table S1. The determination of the binding epitope of C_{44}Mab-6 by ELISA.

Peptide	Coding exon*	Sequence	C_{44}Mab-6
CD44p21–40	2	QIDLNITCRFAGVFHVEKNG	–
CD44p31–50	2	AGVFHVEKNGRYSISRTEAA	–
CD44p41–60	2	RYSISRTEAADLCKAFNSTL	–
CD44p51–70	2	DLCKAFNSTLPTMAQMEKAL	–
CD44p61–80	2/3	PTMAQMEKALSIGFETCRYG	–
CD44p71–90	2/3	SIGFETCRYGFIEGHVVIPR	–
CD44p81–100	3	FIEGHVVIPRIHPNSICAAN	–
CD44p91–110	3	IHPNSICAANNTGVYILTSN	–
CD44p101–120	3	NTGVYILTSNTSQYDTYCFN	–
CD44p111–130	3/4	TSQYDTYCFNASAPPEEDCT	–
CD44p121–140	3/4	ASAPPEEDCTSVTDLPNAFD	–
CD44p131–150	4/5	SVTDLPNAFDGPITITIVNR	–
CD44p141–160	4/5	GPITITIVNRDGTRYVQKGE	–
CD44p151–170	5	DGTRYVQKGEYRTNPEDIYP	–
CD44p161–180	5	YRTNPEDIYPSNPTDDDVSS	–
CD44p171–190	5	SNPTDDDVSSGSSSERSSTS	–
CD44p181–200	5	GSSSERSSTSGGYIFYTFST	–
CD44p191–210	5	GGYIFYTFSTVHPIPDEDSP	–
CD44p201–220	5	VHPIPDEDSPWITDSTDRIP	–
CD44p211–230	5/v3	WITDSTDRIPATSTSSNTIS	–
CD44p221–240	5/v3	ATSTSSNTISAGWEPNEENE	–
CD44p231–250	v3	AGWEPNEENEDERDRHLSFS	+
CD44p241–260	v3	DERDRHLSFSGSGIDDDEDF	–
CD44p251–270	v3/v4	GSGIDDDEDFISSTISTTPR	–
CD44p261–280	v3/v4	ISSTISTTPRAFDHTKQNQD	–
CD44p271–290	v4	AFDHTKQNQDWTQWNPSHSN	–
CD44p281–300	v4	WTQWNPSHSNPEVLLQTTTR	–
CD44p291–310	v4/v5	PEVLLQTTTRMTDVDRNGTT	–
CD44p301–320	v4/v5	MTDVDRNGTTAYEGNWNPEA	–
CD44p311–330	v5	AYEGNWNPEAHPPLIHHEHH	–
CD44p321–340	v5	HPPLIHHEHHEEEETPHSTS	–
CD44p331–350	v5/v6	EEEETPHSTSTIQATPSSTT	–

CD44p341–360	v5/v6	TIQATPSSTTEETATQKEQW	–
CD44p351–370	v6	EETATQKEQWFGNRWHEGYR	–
CD44p361–380	v6	FGNRWHEGYRQTPREDSHST	–
CD44p371–390	v6/v7	QTPREDSHSTTGTAAASAHT	–
CD44p381–400	v6/v7	TGTAAASAHTSHPMQGRTTP	–
CD44p391–410	v7	SHPMQGRTTPSPEDSSWTDF	–
CD44p401–420	v7	SPEDSSWTDFFNPISHPMGR	–
CD44p411–430	v7/v8	FNPISHPMGRGHQAGRRMDM	–
CD44p421–440	v7/v8	GHQAGRRMDMDSSHSTTLQP	–
CD44p431–450	v8	DSSHSTTLQPTANPNTGLVE	–
CD44p441–460	v8	TANPNTGLVEDLDRTGPLSM	–
CD44p451–470	v8/v9	DLDRTGPLSMTTQQSNSQSF	–
CD44p461–480	v8/v9	TTQQSNSQSFSTSHEGLEED	–
CD44p471–490	v9	STSHEGLEEDKDHPTTSTLT	–
CD44p481–500	v9/v10	KDHPTTSTLTSSNRNDVTGG	–
CD44p491–510	v9/v10	SSNRNDVTGGRRDPNHSEGS	–
CD44p501–520	v10	RRDPNHSEGSTTLLEGYTSH	–
CD44p511–530	v10	TTLLEGYTSHYPHTKESRTF	–
CD44p521–540	v10	YPHTKESRTFIPVTSAKTGS	–
CD44p531–550	v10	IPVTSAKTGSFGVTAVTVGD	–
CD44p541–560	v10	FGVTAVTVGDSNSNVNRSLS	–
CD44p551–570	v10/16	SNSNVNRSLSGDQDTFHPSG	–
CD44p561–580	v10/16	GDQDTFHPSGGSHTTHGSES	–
CD44p571–590	16	GSHTTHGSESDGHSHGSQEG	–
CD44p581–600	16/17	DGHSHGSQEGGANTTSGPIR	–
CD44p591–606	17	GANTTSGPIRTPQIPEAAAA	–

+, OD655≧0.5; －, OD655<0.1

*The CD44 exon-cording regions are illustrated in Figure 1.

Supplemental Table S2. Immunohistochemical analysis using C_{44}Mab-6 and C_{44}Mab-46 against colorectal cancer tissues.

No.	Age	Sex	Organ	Pathology diagnosis	Grade	Stage	Type	C_{44}Mab-6	C_{44}Mab-46
1	67	M	Colon	Adenocarcinoma	1	-	Malignant	-	+
2	48	M	Colon	Adenocarcinoma	1	IIA	Malignant	-	-
3	58	M	Colon	Adenocarcinoma	1--2	IIA	Malignant	-	+
4	75	M	Colon	Adenocarcinoma	1	IV	Malignant	-	++
5	86	M	Colon	Adenocarcinoma	2	II	Malignant	-	+
6	55	M	Colon	Adenocarcinoma	2	IIIC	Malignant	-	-
7	38	M	Colon	Adenocarcinoma	1	I	Malignant	-	++
8	52	M	Colon	Adenocarcinoma	1	IIIB	Malignant	-	-
9	46	M	Colon	Adenocarcinoma	2	IIIB	Malignant	+	+
10	61	M	Colon	Mucinous adenocarcinoma	2	IIIB	Malignant	-	++
11	55	M	Colon	Mucinous adenocarcinoma with necrosis	2	IIA	Malignant	-	++
12	55	M	Colon	Adenocarcinoma	1	IIIB	Malignant	-	-
13	44	M	Colon	Adenocarcinoma	1	-	Malignant	-	-
14	31	M	Colon	Adenocarcinoma	2	IIIB	Malignant	-	+
15	74	F	Colon	Adenocarcinoma	2	IIIB	Malignant	+	+
16	61	M	Colon	Adenocarcinoma	2	II	Malignant	+	++
17	45	M	Colon	Adenocarcinoma	2	III	Malignant	-	+
18	58	M	Colon	Adenocarcinoma	2	IIIB	Malignant	-	++
19	58	M	Colon	Adenocarcinoma	2	IIA	Malignant	+	+++
20	69	M	Colon	Adenocarcinoma	3	-	Malignant	-	-
21	64	F	Colon	Adenocarcinoma	2	IIIC	Malignant	+	++
22	82	M	Colon	Adenocarcinoma	2	IIIB	Malignant	-	-
23	34	M	Colon	Adenocarcinoma	2	IIIB	Malignant	-	++
24	50	F	Colon	Adenocarcinoma	2	IIB	Malignant	-	-
25	34	F	Colon	Adenocarcinoma	1	IIB	Malignant	-	+
26	52	F	Colon	Adenocarcinoma	2	IIA	Malignant	-	+
27	53	F	Colon	Adenocarcinoma	2	IIIB	Malignant	-	-
28	58	F	Colon	Adenocarcinoma	2	I	Malignant	-	+
29	59	F	Colon	Adenocarcinoma	2	IIA	Malignant	-	++
30	67	M	Colon	Adenocarcinoma	2	IIIB	Malignant	-	++
31	31	M	Colon	Adenocarcinoma	2	IIIB	Malignant	+	+++
32	54	F	Colon	Adenocarcinoma	2	IIB	Malignant	+	+
33	54	F	Colon	Adenocarcinoma	2	IIIB	Malignant	-	-
34	62	M	Colon	Adenocarcinoma	2	-	Malignant	-	+
35	67	F	Colon	Adenocarcinoma	2	-	Malignant	-	-
36	52	F	Colon	Adenocarcinoma	2	IIA	Malignant	-	-
37	52	F	Colon	Adenocarcinoma	3	IIIB	Malignant	-	-
38	75	M	Colon	Adenocarcinoma	2	-	Malignant	-	-
39	57	F	Colon	Adenocarcinoma	2	IIB	Malignant	-	+++
40	38	M	Colon	Mucinous adenocarcinoma	3	I	Malignant	-	-
